# Fully Printed and Scalable Current and Voltage Sensors for Smart Grid Transmission Line Monitoring

**DOI:** 10.3390/s25072287

**Published:** 2025-04-04

**Authors:** Yanyun Fan, Lei Zhang, Chi Zhang, Zhengang An, Bo Li, Dachao Li

**Affiliations:** State Key Laboratory of Precision Measurement Technology and Instruments, Tianjin University, Tianjin 300072, China; fanyanyun0614@tju.edu.cn (Y.F.); zhangchi0066@tju.edu.cn (C.Z.); anzhengang@tju.edu.cn (Z.A.); lb1185298796@tju.edu.cn (B.L.)

**Keywords:** current sensor, voltage sensor, fully printed, smart grid

## Abstract

In the construction and operation of smart grids, real-time monitoring of electrical signals is crucial for achieving efficient and stable power transmission, so it is necessary to develop current and voltage sensors with high stability, mass manufacturing and light weight. This study presents a current and voltage sensor based on fully printed technology for electrical signal monitoring of transmission lines. The current sensor is supported and insulated by polyimide, and successfully fabricates the 3D induction coil through screen printing and high-precision inkjet printing processes, achieving a sensitivity of 0.00823 mV/A and a linearity of 0.999 in 0–60 A. The voltage sensor is made of polyimide film as the substrate, and a pair of silver sensing electrodes are prepared by screen printing process, achieving a sensitivity of 0.00369 μA/V and a linearity of 0.999 in 0–1200 V, with stable output over a continuous operation of 24 h. The overall size of the current and voltage sensor is 1.5 cm × 2 cm, the weight is 1.8 g, the cost is about USD 0.462, and it has the advantages of low cost, lightweight, good linearity, high stability, simple structure, and scalable preparation. This work provides a new sensor fabrication method for current and voltage monitoring in transmission lines.

## 1. Introduction

With the rapid development of human society and the economy, the power and energy industries have ushered in new developments and changes, forming a new type of power system dominated by renewable energy sources [1,2]. The high proportion of distributed renewable energy connected to the grid and the diversification of power equipment have increased the volatility and uncertainty of the grid, reducing the anti-disturbance ability and adjustment ability of the power grid, which severely impacts the stability of the power system [3,4]. The safe operation of the power grid is not only closely related to the daily life of the people and various industries but also crucial to national economic energy and social security. Therefore, it is essential to monitor, control, analyze, and make decisions on the operation process of smart grids and power equipment to obtain timely status information and deal with abnormal situations [5,6]. However, advanced measurement technologies and sensors are fundamental for obtaining power data.

The measurement of current and voltage is two very important parameters for monitoring, maintaining, and repairing of transmission lines and power equipment, and is widely used in the fields of transmission, transformation, and distribution [7]. For example, a 35 kV substation typically requires around 30 electronic sensors, and the demand for electronic sensors increases with higher voltage levels, and the number of substations with voltage levels of 35 kV and above in China has reached 40,000. Therefore, the power grid system requires a large number of low-cost, lightweight, and easy to install current and voltage sensors. Currently, common current sensors include magnetoresistive current sensors, Hall current sensors, and inductive coil current sensors, all of which determine the measured current by measuring the magnetic field around the conductor. Traditional electromagnetic current transformers are technically mature and cost effective, but have limited measurement ranges, are prone to core magnetic saturation, and are bulky, heavy, and complex to install [8]. Hall current sensor has high linearity, but its volume and weight are generally large and its stability is poor. Inductive coil sensors have a simple structure, low cost, good linearity, can be installed with electricity, have a wide measurement range, and a wide response frequency band, making them more suitable for the measurement needs of power systems [9]. The commonly used voltage sensors are electromagnetic voltage transformer and capacitive voltage transformer. Traditional voltage transformers are bulky and difficult to install due to their complex insulation structure, and are suitable for lower voltage levels [10,11]. Capacitive voltage transformer has a large measurement range, no magnetic saturation problem, and higher insulation strength. Current and voltage sensors can be used to monitor the electrical signal monitoring of transmission lines to ensure the stability and safety of the system operation [12,13,14,15,16].

With the development of MEMS technology, micro electronic sensors have the advantages of small size, light weight, low cost, and conducive to mass production. Watanabe et al. [17] proposed a Rogowski current sensor based on the MEMS process, which uses a silicon via structure to form a helical return coil to eliminate external magnetic field interference, achieving linear current detection in the range of 0–40 A. Yamashita et al. [18] prepared a flexible film current sensor (with 480 turns) using laser drilling and screen printing technology, which can be wrapped around cables to achieve current detection in the range of 0–20 A. Chen et al. [19] prepared two symmetrical sensing electrodes on a flexible substrate using MEMS technology as voltage sensors, showing a sensitivity of 0.38 mV/V in the range of 0–350 V. However, coils are often manually wound or simply machined during the manufacturing process, resulting in uneven winding distribution and turn density, and it is usually a rigid structure, which is difficult to adapt to the limited space or complex shape of the transformer equipment [20]. Printed sensors using printed electronics technology can achieve high-precision patterning on flexible substrates to improve manufacturing efficiency and reduce costs. They can use diverse functional materials, offering more potential for performance optimization of the sensor, and have flexible designs suitable for personalized and small-batch customized production [21]. However, the performance of printing materials is limited by the content of fillers, particle size, and viscosity in inks, so it is necessary to further strengthen the stability of materials and improve the preparation process [22,23].

In this study, we present a fully printed, scalable current and voltage sensor that can be used for electrical signals detection of transmission lines. The current sensor uses polyimide as the support structure and the 3D induction coil is prepared through screen printing and high-precision inkjet printing processes, achieving current detection based on Faraday’s electromagnetic induction law. The current sensor has a sensitivity of 0.00823 mV/A and a linearity of 0.999 within 0–60 A, demonstrating long-term stable operating capability. The voltage sensor uses screen printing technology to prepare two sensing electrodes and detects voltage based on Gaussian law. The voltage sensor has a sensitivity of 0.00369 μA/V in the voltage range of 0–1200 V and a linearity of 0.999, providing stable output over continuous operation for 24 h. The overall size of the current and voltage sensor is 1.5 cm × 2 cm, the weight is 1.8 g, the cost is about USD 0.462. It has the advantages of low cost, light weight, good linearity, good stability, simple structure, large-scale preparation, etc., and can be fixed on the transmission line through a U-shaped device for easy installation.

## 2. Materials and Methods

### 2.1. Materials

Array voltage and current sensors were fabricated using an ultra-precision material deposition system (XPTL Delta Printing System, Wroclaw, Poland) and a commercial screen printing machine (PHP-B Series, Shanghai Hoting Screen Printing Equipment Co., Ltd., Shanghai, China). High viscosity Ag Nanopaste CL85 (viscosity [cp] > 100,000) was purchased from XPTL in Poland for side step printing of sensors, and Ag ink was purchased from Qingdao Nano Print Materials Technology Co., Ltd. (Qingdao, China). is used for screen printing. Both can be used directly without further purification. Permalloy film was purchased from Chenhua Kaichi Nickel Alloy Company (Guangdong, China) as the magnetic core for sensors.

### 2.2. Preparation of Electrical Sensors

The sensor mainly includes flexible substrate, conductive material and magnetic polymer material. Since we need high temperature drying during the printing process, we chose a high-temperature resistant polyimide film as the substrate. Conductive materials use high-viscosity nano silver ink that is matched with high-precision inkjet printing equipment, and other materials may cause print head clogging and uneven printing. Permalloy with high permeability can effectively gather magnetic fields and improve sensor performance.

**Figure 1:**
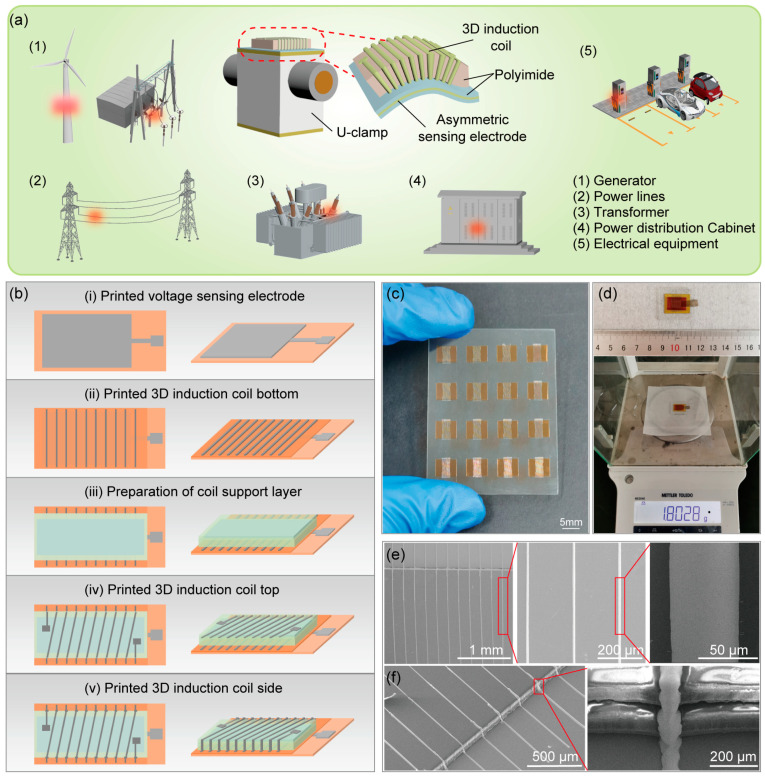
Application and preparation of electronic sensors. (**a**) Schematic diagram of the structure of voltage and current sensors used for monitoring electrical signals on transmission lines. (**b**) The preparation process of current and voltage sensors. (**c**) Optical photo of arrayed electronic sensors. (**d**) Optical photos of electronic sensors size and weight. (**e-f**) SEM images of the 3D induction coil structure of the current sensor.

The preparation process of the current and voltage sensors is shown in Figure 1b. First, a silver electrode is printed on the surface of the polyimide film using screen printing as the voltage sensing electrode, and then dried at 120 °C for 30 min on a heating platform. After drying, a layer of polyimide is adhered to its surface for insulation and as a substrate (Figure 1b-i). Second, the bottom straight of the 3D coil is screen printed on the surface of the polyimide film and dried at 120 °C for 30 min (Figure 1b-ii). Third, a layer of polyimide is first used to cover the bottom of the coil, followed by adhering a layer of permalloy as the magnetic core, and then covering it with a layer of polyimide film. This serves as the support and insulation structure for the coil, ensuring that the polyimide completely wraps the magnetic core to prevent a short circuit between the magnetic core and the coil (Figure 1b-iii). Fourth, print the top oblique line of the 3D coil on the surface of the support structure and dry at 120 °C for 30 min (Figure 1b-iv). Fifth, high-viscosity silver paste is used to print the side of the 3D coil, connecting the silver wires from the bottom and top, forming the 3D induction coil after drying at 220 °C for 15 min (Figure 1b-v).

### 2.3. Characterization and Electrical Measurements

Scanning electron microscopy (SEM) images of the 3D induction coil were observed using a scanning electron microscope (Nanosem 430, FEI, Thermo Fisher Scientific, Hillsboro, OR, USA). An alternating current power supply (ATC70005, Anmtake Electronics, Shenzhen, China) was used to simulate the current and magnetic field conditions of the transmission line, and the induced voltage of the current sensor was detected by an oscilloscope (MDO3, Tektronix, Portland, OR, USA). A high-precision voltage and current source (YOKOGAWA, 2558A, Yokogawa Electric Corporation, Tokyo, Japan) was employed to simulate voltage and current conditions, and the induced current of the voltage sensor was obtained using an editable electrometer (Keithley 6514, Keithley Instruments, Cleveland, OH, USA). A heating platform (JFTOOIS, V-1015, Guangdong, China) was used to change the test temperature of the current and voltage sensors.

### 2.4. Testing of Current and Voltage Sensors

For current sensor testing, the testing platform is shown in Figure S1 (Supporting Information). The alternating current power supply is used to simulate the current of the transmission line, and the sensor is fixed on the surface of the measured wire through the fixture to ensure that the distance between the sensor and the wire is consistent during each test. The outputs at both ends of the sensor are connected to an oscilloscope for recording the peak induced voltage.

For voltage sensor testing, the test system is shown in Figure S2 (Supporting Information). The high precision voltage source is used to simulate the voltage of the transmission line, and the sensor is fixed on both sides of the wire through the designed fixture to ensure that the distance difference between the two electrodes is consistent, in order to improve measurement accuracy. The output current signal of the sensor is collected in real time by an electrometer, and the steady-state current value is recorded.

In order to evaluate the performance of the sensor under different temperatures, a variable temperature test platform is built (Figure S10, Supporting Information). It includes a high-precision voltage and current source, an electrostatic meter, a heating platform, and a variable temperature test device. During the test, the temperature in the device is changed by the heating platform and displayed through a thermometer.

The sensor is fixed using a designed U-shaped clamp, which ensures that the sensor is firmly fixed to the transmission line (Figure S3, Supporting Information), and each installation only needs to remove the U-shaped fixing block, and does not need to remove the sensor position, which avoids damage to the sensor during the installation process. Before each measurement, it is necessary to remove dust and impurities from the surface, check the connection wires’ firmness, and ensure stable signal transmission. When no current passes through, the output of the sensor is zero or close to zero. And each group of tests is repeated 3 times, and the final mean is taken to reduce random errors.

## 3. Results

### 3.1. Configuration and Preparation of Electrical Sensors

The configuration of the electronic sensors includes a voltage sensor, a current sensor, and a custom-printed U-shaped fixture for easy fixation on transmission lines, as shown in Figure 1a. As shown in Figure 1b, the voltage sensor is fabricated by screen printing process to create sensing electrodes on the surface of a polyimide film. The current sensor is based on polyimide as support and insulation structure, and the bottom and top surfaces of the coil are prepared through screen printing, and connected by high-precision inkjet printing technology to form a 3D induction coil. This fully printed fabrication process has the capability for scalable production, as demonstrated in Figure 1c. Figure 1d shows that the size of the current and voltage sensor is 1.5 cm × 2 cm, the weight is 1.8 g, and the cost is about USD 0.462 (Table S1, Supporting Information). Figure 1e illustrates that the surface thickness of the 3D induction coil of the current sensor is uniform, with no contact or interruption between adjacent coils, and the coil surface and bottom surface are successfully connected, with a spacing of 200 μm and a line width of 50 μm. The electrode surface of the voltage sensor is printed uniformly (Figure S4, Supporting Information). As shown in Table S2 (Supporting Information), our sensor performance is compared with previous work. Compared to other sensors, our sensors are flexible, highly linear, and have significant advantages in terms of volume, weight, and cost.

### 3.2. Working Mechanism and Simulation Analysis of Electrical Sensors

#### 3.2.1. Current Sensor

**Figure 2:**
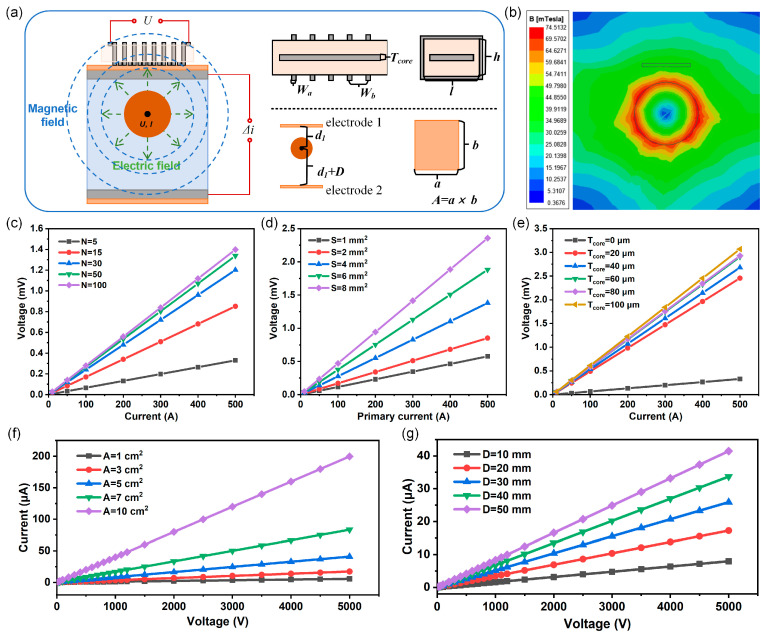
Working mechanism and simulation analysis of electrical sensors. (**a**) Working mechanism of current and voltage sensors. (**b**) Simulated cloud image of the magnetic field around the transmission line (cross-sectional view). (**c**) Simulation of current sensors with different coil turns. (**d**) Simulation of current sensors with different coil cross-sectional areas. (**e**) Simulation of current sensors with different magnetic core thicknesses. (**f**) Simulation of voltage sensors with different sensing electrode areas. (**g**) Simulation of voltage sensors with different distance differences.

The current sensor is based on the Roche coil measurement technology. As shown in Figure 2a, a magnetic field is generated when current through the transmission line, and the magnetic field induces an electromotive force in the coil of the current sensor. According to Faraday’s law of electromagnetic induction [24] the change in current causes a variation in the magnetic flux through the coil, resulting in a change in the induced electromotive force across the sensor, and the measurement of current can be achieved through the Formula (1).

(1) $$U=-N\frac{d\phi }{dt}=-N\frac{\mu_{0}SI}{2\pi r}sin\omega t=-N\frac{\mu_{0}SI}{2\pi r}sin2\pi ft$$

where *U* is the induced electromotive force, *N* is the number of turns of the coil, *Φ* is the magnetic flux through the coil, *μ*_0_ is the vacuum permeability, *S* is the cross-section area of the coil, *I* is the current intensity in the power line, *r* is the distance from the sensor to the surface of the power line, *ω* is the angular frequency of the current, and *f* is the frequency of the current.

Figure 2b shows the simulated magnetic field distribution around the transmission line using Ansoft Maxwell software (21.2.0.), indicating that the magnetic field strength increases as the distance to the conductor decreases. The current sensor is placed on the surface of the transmission line, and as the distance between the sensor and the wire increases, the magnetic field strength gradually weakens, resulting in a decrease in the induced voltage (Figure S5, Supporting Information). Figure 2c,d show the simulation of turns *N* and cross-sectional area *S*, and it can be seen that the induced voltage of the coil gradually increases with the increase of turns and cross-sectional area. Figure 2e shows the effect of the core thickness *T_core_* on the output voltage of the coil, revealing that increasing the magnetic core significantly enhances the coil’s output voltage, while the thickness of the magnetic core has a relatively minor effect on the output voltage. Additionally, simulations of the line width *W_a_* and the distance between lines *W_b_* of the induction coil indicated that the output voltage has almost no relation to line width, with a slight increase as the spacing increases (Figure S5, Supporting Information). Therefore, by increasing the number of turns and cross-sectional area of the 3D induction coil, as well as reducing the distance between the coil and the transmission wire, the sensitivity of the sensor’s current measurement can be improved.

#### 3.2.2. Voltage Sensor

The voltage sensor is based on the capacitive partial voltage measurement technology, which generates different induced current when two electrodes are at different electric field strengths. According to Gauss’s law, the electric field distribution around the transmission line is shown in Figure 2a. The electric field strength is stronger closer to the transmission line and weaker further away from it. The voltage sensor consists of two asymmetric sensing electrodes, and the induced charge in each sensing electrode changes with the variation in voltage, producing induced currents *i_1_* and *i_2_*. The voltage measurement can be realized by the Formula (2).

(2) $$∆i=i_{1}-i_{2}\propto AfUcos\omega t(\frac{1}{d_{1}ln\frac{d_{1}}{R}}-\frac{1}{{(d}_{1}+D)ln\frac{{(d}_{1}+D)}{R}})$$

where Δ*i* is the current difference between electrode 1 and electrode 2, *D* is the distance difference between electrode 1 and electrode 2 to the power line, *d*_1_ and *d*_1_ + *D* are the distance between electrode 1 and electrode 2 to the power line, *A* is the area of the sensing electrode, *f* is the voltage frequency, *U* is the voltage of the power line, *R* is the radius of the power line.

As shown in Figure 2f, as the area of the sensing electrodes increases, more induced charge is generated, resulting in a gradual increase in the output of the sensor. Figure 2g shows that as the distance difference between the two electrodes increases, the output current difference becomes larger, leading to an increase in the output of the sensor. Therefore, the voltage measurement sensitivity of the sensor can be improved by increasing the area of the sensing electrodes and the distance between the two electrodes.

### 3.3. Optimization and Characterization of Electrical Sensors

#### 3.3.1. Current Sensor

**Figure 3:**
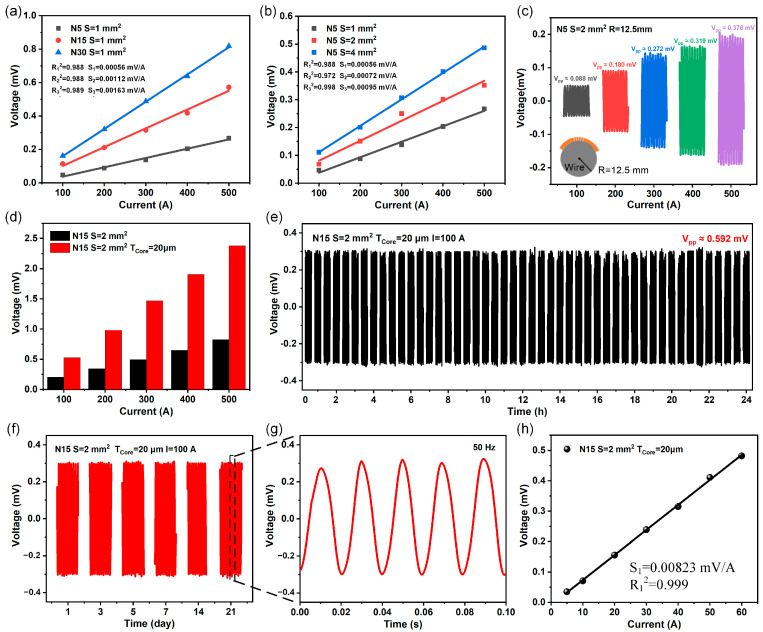
Optimization and characterization of current sensors. (**a**,**b**) The output of current sensor with different turns and cross-sectional areas relative to the current intensity in the power line. (**c**) The output of the current sensor is under a certain degree of curvature. (**d**) Comparison of the output of the current sensor before and after the addition of the magnetic core. (**e**) Cycle characteristics of the current sensor during 24 h of continuous operation. (**f**) Stability of the current sensor after 21 days of operation. (**g**) The output signal of the current sensor is at 50 Hz. (**h**) Output of the current sensor in the range of 0–60 A.

We optimize the structure of the current sensor from the number of coil turns (*N*), cross-sectional area (*S*), and magnetic core (*T_Core_*), and prepare a series of 3D inductive coils with different structures. The sensor is fixed on the surface of the wire being tested, the output signal is displayed through an oscilloscope, and the performance of the current sensor with different structures is tested by applying the current of 50 Hz and 100–500 A. First, we studied the effect of the number of coil turns and cross-sectional area on the output performance. As shown in Figure 3a, the output of the sensor increases with the increase of turns, the output increases from 0.267 mV to 0.818 mV at 500 A. Figure 3b shows that with the increase of cross-sectional area of the coil, the output increases from 0.267 mV to 0.486 mV at 500 A. With the increase of turns and cross-sectional area, the sensitivity and linearity of the sensor are improved, and the detailed experimental data provide strong support for the optimization design (Figure S6, Supporting Information). A greater number of turns and a larger cross-sectional area result in a higher magnetic flux through the coil, leading to increased output, although this also makes fabrication more challenging. Subsequently, we studied that the sensor could maintain stable output under bending while exhibiting a certain degree of flexibility, as shown in Figure 3c. Then, considering that a magnetic core can enhance the magnetic gathering effect, we compared the output of the sensor before and after adding the magnetic core. Figure 3d shows that the output significantly increased after the addition of the magnetic core, which increased by about three times.

Based on the fabrication process, we select a sensor with 15 turns, a cross-sectional area of 2 mm^2^, and a magnetic core thickness of 20 μm for further performance characterization. Since the sensor requires continuous operation for extended periods, we conduct both short-term and long-term stability tests at the temperature of ~22 °C and the relative humidity maintained of ~45%. As shown in Figure 3e,f, the sensor shows good stability during continuous 24 h test, and also maintains a constant output after 21 days of long-term operation. Figure 3g shows that the frequency of the sensor output signal is consistently 50 Hz. In order to accurately analyze the noise level of the sensor, the signal-to-noise ratio (SNR) of the current sensor is analyzed. The SNR values of the current sensor are all over 30 dB, indicating that the noise has relatively little influence on the sensor (Figure S8, Supporting Information). In addition, we test the current from 0–60 A using a high-precision current source. Figure 3h shows that when the current is 60 A, the output voltage is 0.482 mV, the sensitivity is 0.00369 μA/V, and the linearity can reach 0.999. When the current range is 100–500 A, the output of the sensor can reach 2.329 mV (Figure S6, Supporting Information). In the experiment, the temperature increased from 22 °C to 40 °C, the outputs of the current sensor remained stable, indicating that the sensor has good temperature adaptability.

#### 3.3.2. Voltage Sensor

We optimize the performance of the voltage sensor from the area of the sensing electrode (*A*) and the distance difference between the two electrodes (*D*), and prepare a series of voltage sensors with different structures. The two electrodes are asymmetrically fixed on either side of the power line, and the output signal at both ends of the sensor is displayed using an electrometer, and the performance is tested by applying a voltage of 50 Hz and 0–1200 V.

**Figure 4:**
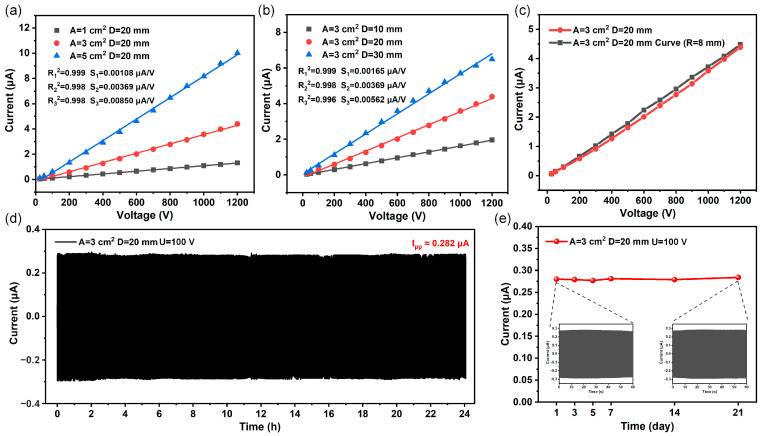
Optimization and characterization of voltage sensors. (**a**,**b**) The output of voltage sensor with different electrode areas and distance differences relative to the voltage intensity in the power line. (**c**) Comparison of the output of the voltage sensor before and after bending. (**d**) Cycle characteristics of the voltage sensor during 24 h of continuous operation. (**e**) Stability of the voltage sensor after 21 days of operation.

First, Figure 4a shows that with the area of the sensing electrodes increases, the output of the sensor increases from 1.31 μA to 10.02 μA at 1200 V. Figure 4b shows that as the distance between the two sensing electrodes increases, the output increases from 1.96 μA to 6.49 μA at 1200 V. Voltage sensors with different structures have excellent linearity, with R^2^ reaching above 0.998. In addition, the larger the sensor area, the higher the sensitivity, which can reach 0.00850 μA/V, and the experimental data are shown in Figure S7 (Supporting Information). The larger the area and distance difference of the sensing electrode, the greater the induced charge difference generated, resulting in a higher output, but this also increases the size of the sensor. Considering the balance between volume and sensing performance, we conduct further performance characterization using a voltage sensor with electrode areas of 3 cm^2^ and a distance difference of 20 mm. Subsequently, we study the output of the sensor when bent, and Figure 4c indicates that its output remains nearly consistent at the curvature radius of 8 mm, reaching 4.4 μA with a sensitivity of 0.00369 μA/V, which can effectively fit transmission lines. Then, we perform short-term and long-term stability tests on the sensor with the temperature of ~22 °C and the relative humidity maintained of ~45%, as shown in Figure 4d,e. The output of the sensor remained constant after a continuous 24 h operation and after 21 days of work, demonstrating excellent stability and long-term operational capability. Over a 21-day period, the average output current is 0.280 μA, with a maximum fluctuation difference of 0.004 μA, which is only 1.4%. As shown in Figure S9 (Supporting Information), the SNR values of the voltage sensors all exceed 40 dB, indicating that the signal quality measured by the sensors is relatively high. In addition, the response of the voltage sensor at different temperatures is tested, showing stable output from 22 °C to 40 °C (Figure S12, Supporting Information).

## 4. Conclusions

In conclusion, we propose a fully printed process to manufacture lightweight, low-cost, and structurally simple current and voltage sensors for monitoring electrical signals in transmission lines. The integrated current and voltage sensor has an overall size of 1.5 cm × 2 cm, the weight is 1.8 g, the cost is about USD 0.462, demonstrating a unique lightweight design and economic benefits. In addition, customized U-shaped fixtures can be non-destructively installed on transmission lines of different sizes, with wide applicability. Among them, the current sensor is based on Rogowski coil measurement technology, in the 0–60 A measurement range, the highest output can reach 0.482 mV, with high sensitivity of 0.00823 mV/A, excellent linearity of 0.999, and excellent signal-to-noise ratio more than 30 dB. The voltage sensor is based on the principle of thin-film capacitive voltage division, with a sensitivity of 0.00369 μA/V in the range of 0–1200 V, with linearity of 0.998 and signal-to-noise ratio up to 40 dB. The current and voltage sensors show excellent long-term stability, with an output fluctuation rate of less than 1.4% after 21 days of continuous operation. Therefore, sensors prepared using full printing manufacturing technology have the characteristics of flexibility, high-precision patterning, and small batch customized production, which is conducive to the preparation of sensors with small size, light weight, and low cost, providing a new technical method for high-density and low-cost deployment of intelligent node monitoring in distributed smart grids.

## Data Availability

Data are contained within the article and Supplementary Materials.

## Supplementary Materials

The following supporting information can be downloaded at: https://www.mdpi.com/article/10.3390/s25072287/s1, Figure S1: (a) Current sensor test platform, (b) test block diagram. Figure S2: (a) Voltage sensor test platform, (b) test block diagram. Figure S3: (a,b) Photos of sensors fixed on different wires using U-shaped fixtures. Figure S4: SEM image of electrode surface of voltage sensor. Figure S5: Simulation of current and voltage sensors. (a) Simulation model of the current sensor. (b) Simulation model of the voltage sensor. (c–e) Simulation images show the variation of current with different distances between the sensor and the wire, different line widths of the sensor, and different distances between the lines. Figure S6: Performance test of current sensors with different structures. (a–h) Output of 3D induction coils with turns of 5, 15, and 30, and cross-sectional areas of 1 mm^2^, 2 mm^2^, and 4 mm^2^ in the range of 100–500 A. (i) Output of a current sensor in the range of 100–500 A. Figure S7: Performance test of voltage sensors with different structures. (a–c) Output of voltage sensors with electrode areas of 1, 3, and 5 cm^2^ in the range of 0–1200 V. (d–f) Output of voltage sensors with a distance difference of 10, 20, and 30 mm between the two electrodes in the range of 0–1200 V. Figure S8: Noise analysis of current sensors. Figure S9: Noise analysis of voltage sensors. Figure S10: Variable temperature test platform. Figure S11: The output of the current sensor varies with temperature. Figure S12: The output of the voltage sensor varies with temperature. Table S1: The cost of an electronic sensor with N = 15, S = 2 mm^2^, and A = 3 cm^2^. Table S2: Performance comparison of current and voltage sensors.
