# Supplementary material for: Fully Printed and Scalable Current and Voltage Sensors for Smart Grid Transmission Line Monitoring
Source: Sensors (Basel). 2025 Apr 4;25(7):2287. doi: 10.3390/s25072287 (PMC11990999; doi:10.3390/s25072287)
Supplement: Supplementary file 1 [file sensors-25-02287-s001.zip › sensors-3533850-supplementary.pdf]

Supporting Information

Fully Printed and Scalable Current and Voltage Sensors for Smart Grid Transmission Line Monitoring

Yanyun Fan †, Lei Zhang *,†, Chi Zhang, Zhengang An, Bo Li and Dachao Li *

State Key Laboratory of Precision Measurement Technology and Instruments, Tianjin University, Tianjin 300072, China

* Correspondence: zhangleitd@tju.edu.cn (L.Z.); dchli@tju.edu.cn (D.L.)

† These authors contributed equally to this work.

Content of the Supplementary Information

Figure S1 (a) Current sensor test platform, (b) test block diagram.

Figure S2 (a) Voltage sensor test platform, (b) test block diagram.

Figure S3 (a, b) Photos of sensors fixed on different wires using U-shaped fixtures.

Figure S4 SEM image of electrode surface of voltage sensor.

Figure S5 Simulation of current and voltage sensors. (a) Simulation model of the current sensor. (b) Simulation model of the voltage sensor. (c–e) Simulation images show the variation of current with different distances between the sensor and the wire, different line widths of the sensor, and different distances between the lines.

Figure S6 Performance test of current sensors with different structures. (a–h) Output of 3D induction coils with turns of 5, 15, and 30, and cross-sectional areas of 1 mm², 2 mm², and 4 mm² in the range of 100–500 A. (i) Output of a current sensor in the range of 100–500 A.

Figure S7 Performance test of voltage sensors with different structures. (a–c) Output of voltage sensors with electrode areas of 1, 3, and 5 cm² in the range of 0–1200 V. (d-f) Output of voltage sensors with a distance difference of 10, 20, and 30 mm between the two electrodes in the range of 0–1200 V.

Figure S8 Noise analysis of current sensors.

Figure S9 Noise analysis of voltage sensors.

Figure S10 Variable temperature test platform.

Figure S11 The output of the current sensor varies with temperature.

Figure S12 The output of the voltage sensor varies with temperature.

Table S1 The cost of an electronic sensor with N=15, S=2 mm², and A=3 cm².

Table S2 Performance comparison of current and voltage sensors.

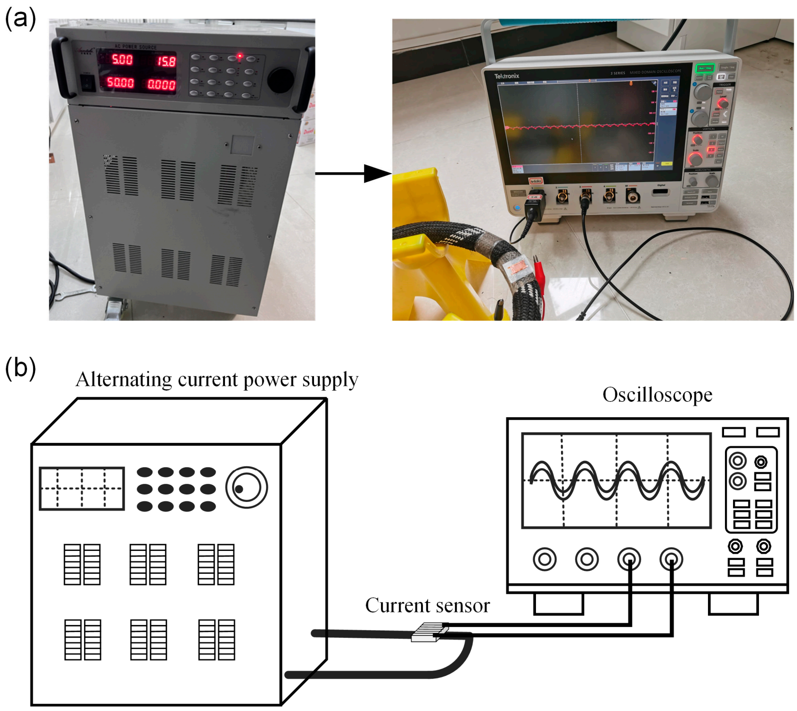

Figure S1 (a) Current sensor test platform, (b) test block diagram.

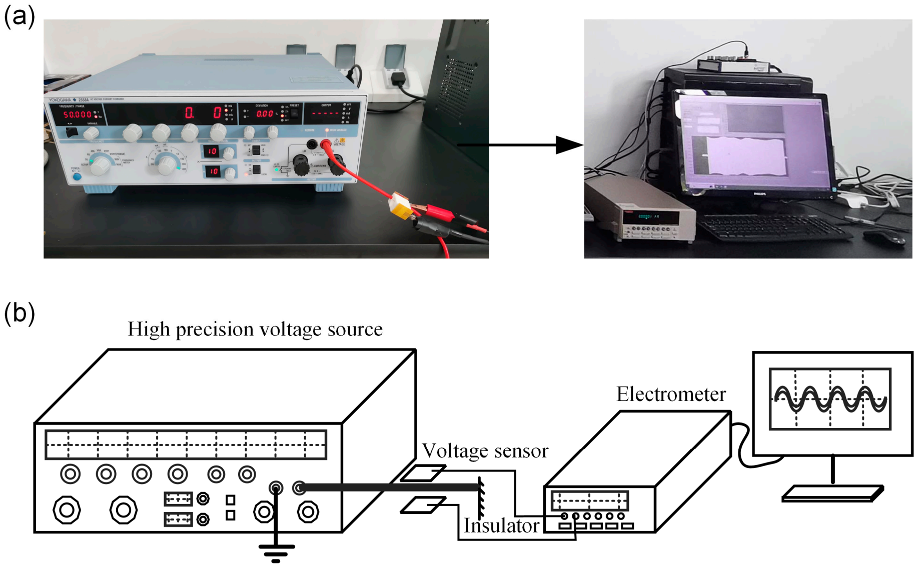

Figure S2 (a) Voltage sensor test platform, (b) test block diagram.

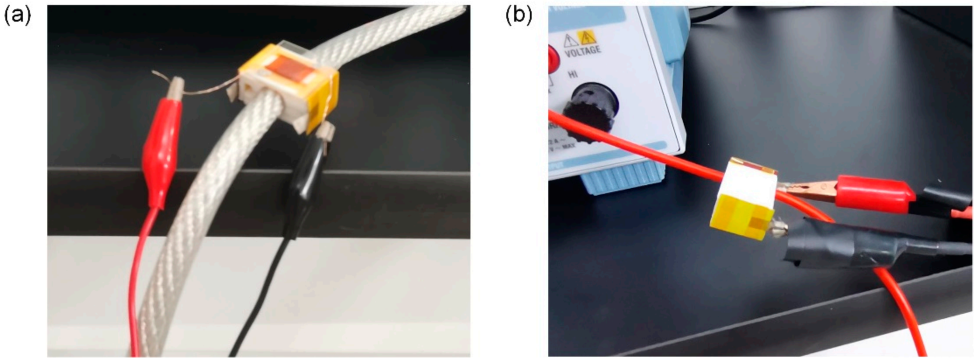

Figure S3 (a, b) Photos of sensors fixed on different wires using U-shaped fixtures.

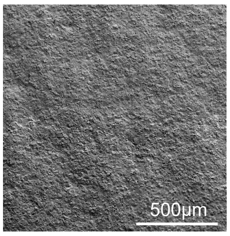

Figure S4 SEM image of electrode surface of voltage sensor.

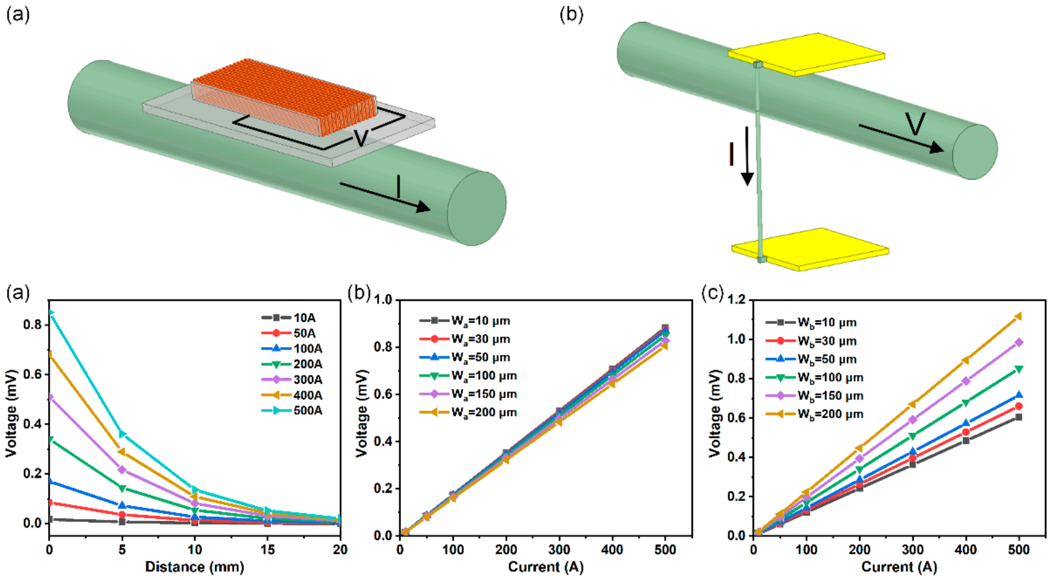

Figure S5 Simulation of current and voltage sensors. (a) Simulation model of the current sensor. (b) Simulation model of the voltage sensor. (c–e) Simulation images show the variation of current with different distances between the sensor and the wire, different line widths of the sensor, and different distances between the lines.

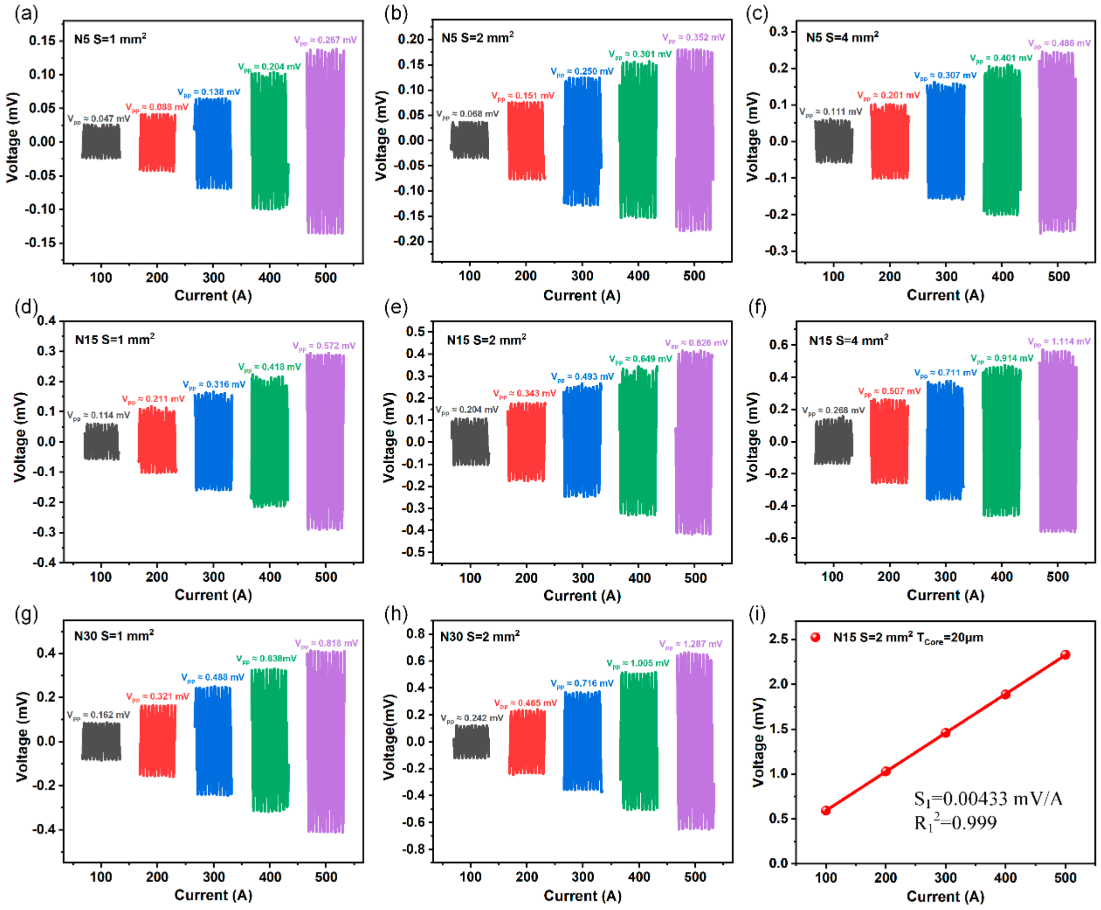

Figure S6 Performance test of current sensors with different structures. (a-h) Output of 3D induction coils with turns of 5, 15, and 30, and cross-sectional areas of 1 mm², 2 mm², and 4 mm² in the range of 100-500 A. (i) Output of a current sensor in the range of 100-500 A.

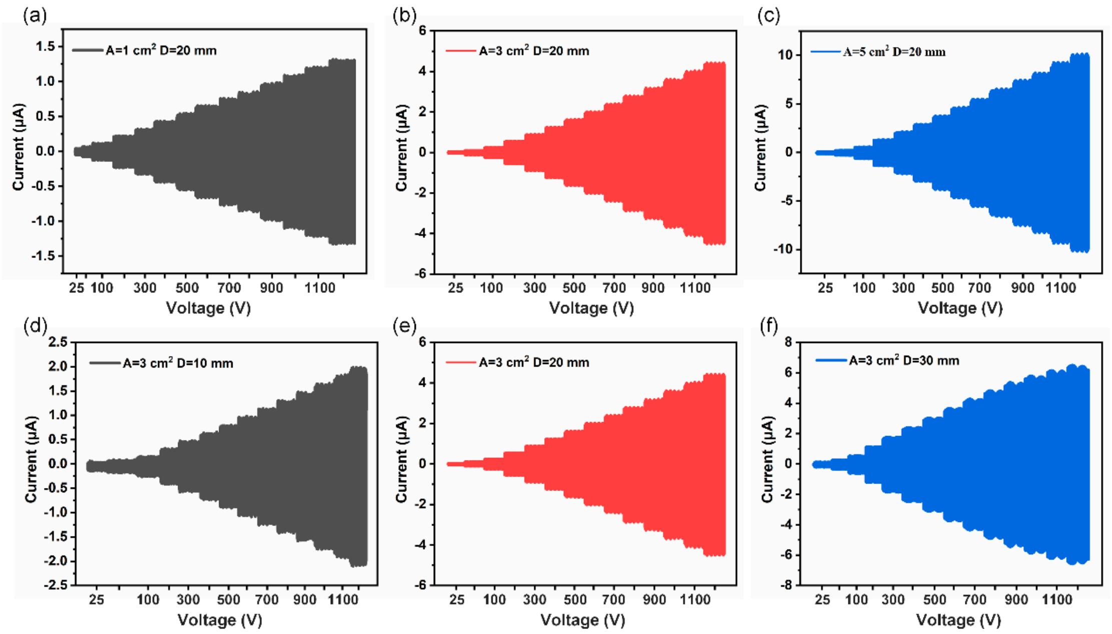

Figure S7 Performance test of voltage sensors with different structures. (a–c) Output of voltage sensors with electrode areas of 1, 3, and 5 cm^2 in the range of 0–1200 V. (d-f) Output of voltage sensors with a distance difference of 10, 20, and 30 mm between the two electrodes in the range of 0-1200 V.

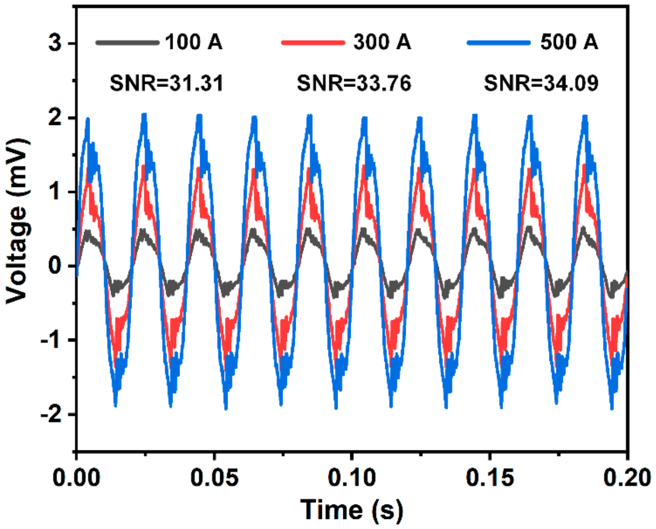

Figure S8 Noise analysis of current sensors.

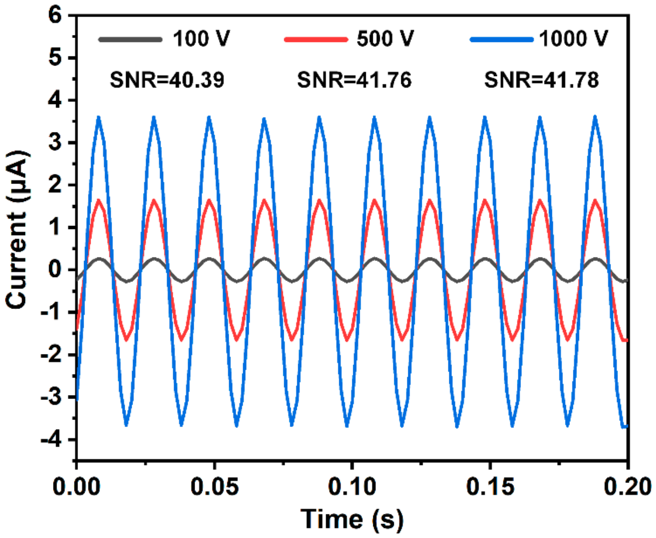

Figure S9 Noise analysis of voltage sensors.

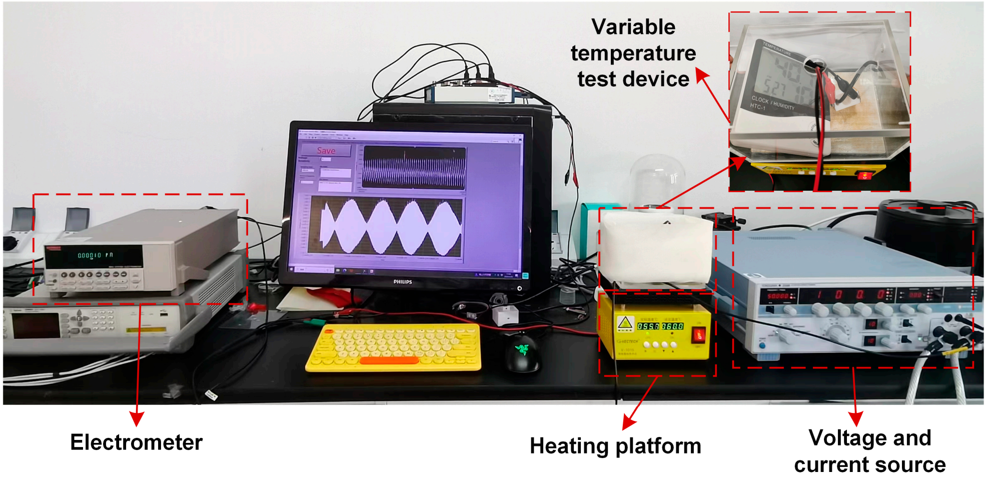

Figure S10 Variable temperature test platform.

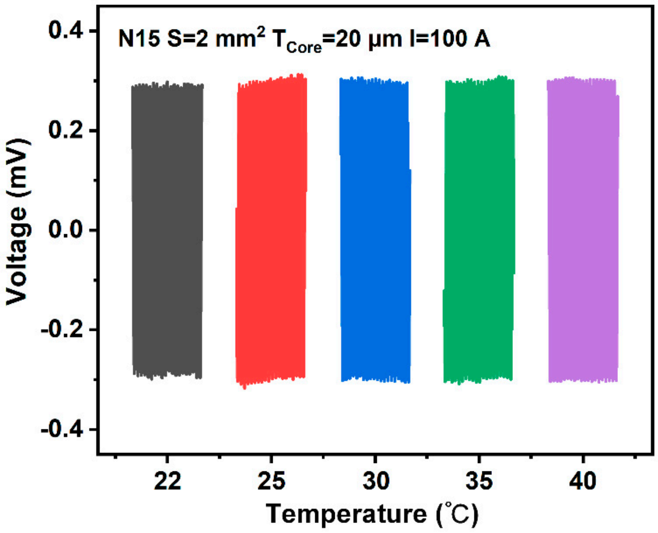

Figure S11 The output of the current sensor varies with temperature.

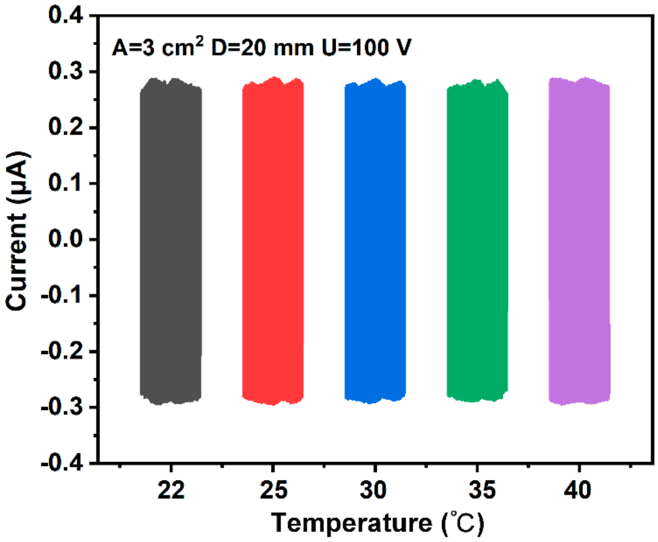

Figure S12 The output of the voltage sensor varies with temperature.

Table S1 The cost of an electronic sensor with N=15, S=2 mm^2, and A=3 cm^2.

Material	Unit price (USD)	Consumption	Cost (USD)
Polyimide film	0.0685/cm^2	5.5 cm^2	0.3768
Ag ink	1.09/ml	0.0744 ml	0.0811
Ag Nanopaste	4.56/ml	0.000015 ml	0.0001
Permalloy film	0.0082/cm^2	0.3 cm^2	0.0025
Ag adhesive tape	0.0308/m	4 cm	0.0012
Total			0.4617

Table S2 Performance comparison of current and voltage sensors.

References	Measuring principle	Measuring Range (sensitivity, linearity)	Size/mm	Weight	Cost (USD)
Our work	Rogowski coil, Capacitive voltage transformer	0-60 A (0.00823 mV/A, 0.999)<br>100-500 A (0.00433 mV/A, 0.999)<br>0-1.2 kV (0.00369 μA/V, 0.998)	20×15	1.8 g	0.462
CSNX1000M	Hall effect	±1500 A (0.999)	110×95×34	/	107.5
LA55-P/SP23	Hall effect	±70 A (0.998)	37×27×14	18 g	40.0
LTS6-NP	Hall effect	±20 A (0.104 V/A, 0.999)	24×22×11	10 g	9.6
RS-14-1KA	Rogowski coil	10 mA–1 kA (0.1 V/A)	56×48×30	500 g	363.7
DVL 1000	Voltage sensor	±1500 V (50 μA/V, 0.995)	138×63×64	290 g	199.8
